# Safety of Onabotulinumtoxin-A for Chronic Migraine During Pregnancy and Breastfeeding: A Narrative Review

**DOI:** 10.3390/toxins17040192

**Published:** 2025-04-11

**Authors:** Antonio Russo, Luigi Francesco Iannone, Ilaria Orologio, Veronica Rivi, Alberto Boccalini, Flavia Lo Castro, Marcello Silvestro, Simona Guerzoni

**Affiliations:** 1 Antonio Russo Headache Center, Department of Advanced Medical and Surgical Sciences, University of Campania “Luigi Vanvitelli”, 80138 Naples, Italymarcello.silvestro6@gmail.com (M.S.); 2 MRI Research Center SUN-FISM, University of Campania “Luigi Vanvitelli”, 80138 Naples, Italy; 3 Digital and Predictive Medicine, Pharmacology and Clinical Metabolic Toxicology-Headache Center and Drug Abuse-Laboratory of Clinical Pharmacology and Pharmacogenomics, AOU Policlinico Di Modena, 41124 Modena, Italyveronica.rivi@unimore.it (V.R.);; 4 Department of Biomedical, Metabolic, and Neuroscience Science, University of Modena and Reggio Emilia, 41125 Modena, Italy

**Keywords:** Onabotulinumtoxin-A, pregnancy, breastfeeding, chronic migraine, safety, fetal outcomes

## Abstract

Onabotulinumtoxin-A (onabotA) is a neurotoxin widely used for several indications, including chronic migraine (CM) preventive treatment, due to its well-demonstrated efficacy, tolerability, and safety. However, onabotA safety during pregnancy and breastfeeding remains unclear, as these populations are typically excluded from clinical trials. The action of onabotA starts locally at the injection sites, modulating the pain pathway with minimal systemic absorption, which theoretically minimizes risks to the fetus or breastfeeding infant. Preclinical studies demonstrate that onabotA does not distribute systemically in significant amounts after administration, although adverse fetal outcomes in rats and rabbits were reported when injected at high doses. Limited human data suggest that onabotA exposure during pregnancy may not be associated with major malformations or significant adverse outcomes for the fetus, especially when used at therapeutic doses for migraine prevention during the first trimester or earlier. Data on breastfeeding are even scarcer but indicate a low likelihood of drug transfer into breast milk. This narrative review highlights the available evidence on the use of onabotA in pregnancy and breastfeeding women, including real-word evidence, with a focus on the use for CM.

## 1. Introduction

Migraine is a complex neurological disease characterized by recurrent and disabling attacks of headache associated with neurovegetative symptoms such as nausea and vomiting and sensory hypersensitivity with photo/phonophobia [1,2].

It affects approximately 12% of the global population and is burdened by physical, emotional, and economic impacts [3]. In its chronic form, migraine is defined as headaches occurring on at least 15 days per month with at least eight of these fulfilling the criteria for migraine. Chronic migraine (CM) typically derives from episodic migraine as the result of increasing attack frequency in response to several risk factors that are known to be implicated with migraine chronicization [4]. People with CM, about 2–4% of the general population, are characterized by higher disability and incidence of comorbidities in comparison to people with episodic migraine, such that CM has been ranked as the first cause of disability among women of childbearing age [3].

The therapeutic approach for migraine relies on acute (as needed) and preventive treatments [5]. Traditionally, preventive treatments for migraine were exclusively represented by oral drugs (so-called repositioning drugs) used for other disorders that were found to be effective in the treatment of migraine by serendipity, such as antiseizure medications (ASMs), beta-blockers, and antidepressants [6]. In the last decade, migraine preventives have been significantly widened by the approval of effective and well-tolerated drugs such as Onabotulinumtoxin-A (onabotA) [7], and, more recently, the inhibitors of the calcitonin gene-related peptide (CGRP) pathway (both monoclonal antibodies and small molecules, the latter of which are known as gepants) [8].

Nevertheless, the therapeutic approach to migraine is a relevant problem during pregnancy and breastfeeding since oral standard of care drugs are limited or prohibited (i.e., valproic acid or topiramate) due to their potential teratogenic effects or adverse outcomes on fetal development [9,10]. In particular, the management of prophylactic medications during pregnancy is challenging because, based on the safety profile of different drugs, there are several limitations. Beta-blockers (e.g., metoprolol and propranolol) and magnesium are frequently used and remain the first choice during pregnancy. However, in using magnesium, transient neurological symptoms and hypotonia in neonates have been reported, and, if magnesium is administered intravenously over a long time, bone abnormalities are described [11]. Potential fetal side effects, like intrauterine growth retardation, preterm birth, and respiratory distress, are also reported. It is advisable to taper/stop beta-blockers a few days before birth to limit the possibilities of neonatal bradycardia and uterine contractions. Low-dose tricyclic antidepressants, such as amitriptyline, are considered safe to use during pregnancy, although some studies suggest a possible teratogenic effect (cardiovascular or limb defects). Antiepileptic drug use during pregnancy is not recommended. Valproate is contraindicated because of serious fetal side-effects like neural tube defects and other major malformations such as cleft palate or cardiac or urinary tract defects; topiramate during pregnancy can increase the risk of cleft lip/palate and low birth weight [11,12].

Unfortunately, onabotA and anti-CGRP drugs have not been specifically tested in pregnancy or breastfeeding women, as it is a population excluded from randomized controlled trials (RCTs) and largely from real-world studies. Therefore, onabotA was labeled as an FDA category C drug (meaning that risks cannot be ruled out) during pregnancy. In 2015, the Food and Drug Administration (FDA) system for the use of drugs in pregnancy and lactation removed any references to categories A, B, C, D, and X and replaced them with a summary of the perinatal risks of the substance [13].

In principle, the key role of CGRP in fetal development, placental function, and vascular regulation raises concerns about the risk of systemically inhibiting its activity by using anti-CGRP monoclonal antibodies and gepants, although no safety signals have been reported for both classes so far, considering registrative studies and real-word experiences [14,15].

On the other hand, onabotA, locally administered (intramuscularly) for chronic migraine (CM) treatment according to the PREEMPT protocol (fixed-site and -dose injections into seven head and neck muscle areas) [16] and due to its minimal systemic absorption (see pharmacological consideration paragraph below), is suggested to have a very low risk to the fetus during pregnancy or to newborn during breastfeeding. Based on these foregoing factors, the recent International Headache Society practical recommendation for preventive migraine treatment tends to suggest onabotA for women with chronic migraine during pregnancy and breastfeeding, representing an option after balancing risks and benefits given the limited systemic effects [17]. The present narrative review aims to summarize the clinical evidence (including more than 450 exposures) on onabotA for CM management in pregnant and breastfeeding women, highlighting safety and tolerability, to provide an easily consultable, comprehensive overview for clinical practice.

## 2. Results

The majority of studies reported the use of onabotA for other indications than migraine. In this review we reported data from all eligible studies, exclusively focusing on onabotA as preventive treatment in women with CM, as detailed in Table 1. Overall, evidence rely on case reports, case series and observational studies (mainly retrospective).

### 2.1. Studies Including OnabotA for the Treatment of Chronic Migraine During Pregnancy and Breastfeeding

The first report on the use of onabotA for CM treatment during pregnancy was the case of a 26-year-old woman who restarted onabotA during gestation and experienced significant migraine relief with no adverse pregnancy outcomes [18]. The patient was treated with onabotA before pregnancy with clinically significant benefits but, when she stopped before pregnancy, she experienced a significant worsening of migraine-attack frequency and the accompanying symptoms. Although informed about the potential risks, the patient restarted onabotA at the 18th week at a dose of 71 U, which was significantly lower than the 155–195 U of the PREEMPT protocol. Unfortunately, no information is reported on the injection paradigm adopted to inject the 71 U of onabotA. Nevertheless, once again, the treatment with onabotA resulted in a significant reduction in the frequency and intensity of migraine attacks. Interestingly, a follow-up of the child’s medical history from birth to age 6 revealed normal neuromuscular development, with all growth milestones met as expected.

A cumulative 29-year safety update [19] on pregnancy outcomes in 397 patients exposed to onabotA treatment, mainly for aesthetic purposes (35.3%) or for CM treatment (30.3%), followed 195 prospective pregnancies (with 197 fetuses), resulting in 152 live births (77.2%) and 45 fetal losses (22.8%). More specifically, 94.6% of onabotA exposures occurred before conception or during the first trimester of pregnancy, with the dose being less than 200U in the majority of patients, and nearly 46% of the women were aged 35 or older. Among the 45 fetal losses (22.8%), 32 were spontaneous miscarriages and 13 were elective abortions. Among the 152 live births, 148 (97.4%) newborns had normal outcomes, while four (2.6%) newborns had abnormal outcomes. Surprisingly, the overall fetal defects rate in the live births was 2.6% (0.7% were major fetal defects), which is lower than the expected general population’s rate for fetal defects of 3–6%.

Overall, the study results suggest that onabotA exposure during pregnancy may not be associated with major birth defects when exposure occurs before conception or early in pregnancy and at lower doses.

In 2020, Wong and Colleagues [20] reported the first prospective real-world study on 45 patients who became pregnant while receiving onabotA treatment. While all patients received onabotA within 3 months prior to conception; only 32 decided to continue treatment during pregnancy with quarterly administration of 155–195 onabotA units, according to the PREEMPT protocol. Among the latter, 31 delivered full-term, healthy babies with no congenital malformations. Only one patient experienced a miscarriage at nine gestational week, which was followed by another pregnancy within the following 4 months that was delivered at full-term without any complications. These results might suggest that neither the exposure within 3 months prior to conception nor continuing onabotA during the whole pregnancy period are associated with adverse pregnancy outcomes, such as congenital abnormalities or low birth weight. In the group of 13 patients who discontinued onabotA, all of them had full-term deliveries, with one forceps-assisted delivery (without negative outcomes). Notably, among patients who stopped treatment, due to the worsening of their migraines by the 4th to 6th month after discontinuation, nine chose to resume onabotA after pregnancy.

These data indicate that onabotA treatment is not associated with an increased risk of major fetal defects, although data availability on exposure during the second and third trimesters is still limited to date. However, given both the potential risks and the lack of extensive studies, such a decision should be carefully made on a case-by-case basis, considering the severity of the mother’s migraine condition and the available alternatives and after balancing the risks and benefits and informing the person of the potential secondary effects and associated risks. 

### 2.2. Studies Including OnabotA for the Treatment of Other Conditions During Pregnancy or Breastfeeding

As mentioned before, onabotA is indicated for the treatment of different conditions (i.e., overactive bladder with symptoms of urge urinary incontinence, urgency, and frequency; urinary incontinence due to detrusor overactivity associated with a neurologic condition; neurogenic detrusor overactivity in pediatric patients five years of age and older; cervical dystonia in adult patients to reduce the severity of abnormal head position and neck pain; severe axillary hyperhidrosis that is inadequately managed by topical agents in adult patients; blepharospasm associated with dystonia in patients 12 years of age and older; and strabismus in patients 12 years of age and older), as well as for aesthetic purposes [21,22].

To date, there is only one specific study that has evaluated the use of onabotA during pregnancy [19]; therefore, case reports and case series are the main source of information about onabotA in pregnancy (see undermentioned studies).

It is worthwhile to mention the study conducted on four women using onabotA (with a total of 40–95 UI) for cosmetic indications that, without breastfeeding, collected breast milk at four different timepoints (2 h after treatment and then on days 2, 3 and 5) to be analyzed [23]. OnabotA was detectable using an enzyme-linked immunosorbent assay (ELISA) kit in two patients (8 out of 16 total samples), with the levels of onabotA ranging from 85.24 to 746.82 pg/mL, which is an amount considered below the reported lethal dose for infants.

#### 2.2.1. Studies Including OnabotA for the Treatment of Cervical Dystonia During Pregnancy

Newman and colleagues first reported the case of a 26-year-old woman, suffering from idiopathic cervical dystonia, who experienced four uncomplicated pregnancies while receiving treatments with onabotA (from 600 up to a maximum of 1200 total units per pregnancy) from the pre-conception period until to the end of gestation. None of the children were breastfed. Although the children were not examined directly, the mother did not report any delays in cognitive or motor development during a follow-up period up to 5 years [24]. In another case series, two patients received onabotA (200 or 500 units) only during the first trimester of pregnancy. The first patient, a 38-year-old woman, received 200 units of onabotA at 2 weeks of pregnancy and she delivered at full term a healthy baby. Contrariwise, the second patient, a 39-year-old woman, received onabotA 500 UI during the first month of gestation but two weeks after the injection, at the 10-week point of the twin pregnancy, the heartbeat was not detectable. Nevertheless, a causative relation between the onabotA injection and the miscarriage cannot be established, as the patient had several risk factors, including age, concomitant medications, twin gestation, and a previous history of miscarriage [25]. Finally, the most recent case report is of a patient injected with 250 units of onabotA, twice during the pregnancy (once in the second trimester and once in the third), delivered a healthy baby at 40 weeks’ gestation with no adverse pregnancy outcomes [26].

#### 2.2.2. Studies Including OnabotA for the Treatment of Strabismus During Pregnancy

Only one case described a 17-year-old female treated with 2.5 units of onabotA for a left convergent squint; she found out three days after the second onabotA injection that she was pregnant. No adverse effects were described for either the mother or the child [27].

#### 2.2.3. Studies Including OnabotA for Aesthetic Medicine Procedures During Pregnancy

A case series described two patients who inadvertently received onabotA early in the first trimester for cosmetic purpose. The first, a 34-year-old patient, received 54 units to treat periorbital lines while she was 6 weeks pregnant, while the second woman, a 37-year-old woman, received 65 units to treat facial dynamic lines, unaware that she was 5 weeks pregnant. No adverse events for either mother or child were reported in both cases [28].

Similarly, another short report described a 46-year-old healthy patient who required cesarean section under general anesthesia for worsening severe pregnancy-induced hypertension and hemolysis, elevated liver enzymes, and low platelet count (i.e., HELLP syndrome), who received multiple bilateral cosmetic facial onabotA injections in the first trimester of pregnancy. However, due to lack of information about the mother’s clinical history, as well as the dose of onabotA received and the cause–effect time relation, no relationship between the onabotA treatment and the registered adverse events was drawn [29].

#### 2.2.4. Miscellaneous

In a survey of 396 neurologists conducted by Morgan and colleagues, 12 physicians (3% of overall responders) injected onabotA in 16 pregnant women, mostly during the first trimester of gestation. The doses used ranged from 1.25 to 300 units, and the indications were as follows: cervical dystonia in nine patients; strabismus and blepharospasm in two patients each; and limb dystonia, oromandibular dystonia, and spasmodic dysphonia in one patient each. Only one patient (treated with 300 UI for cervical dystonia), with a previous history of spontaneous abortions, experienced a miscarriage, while another woman underwent a therapeutic abortion. All other pregnancies reached the term, with no reported negative fetal outcomes. Based on the data coming from the survey of treating physicians in the USA, although burdened by the small sample size, onabotA appears to be relatively safe for both pregnant women and their fetuses [30].

Three cases of esophageal achalasia treated during pregnancy with intra-esophageal injection of onabotA were described by three different case reports, with no complications reported for the mothers or the newborns [31,32,33]. Cardon and colleagues described a small case series of three patients who underwent intralaryngeal injection of onabotA during pregnancy for adductor spasmodic dysphonia, with no adverse events for either the mothers or fetuses [34].

## 3. Pharmacological Consideration in Pregnancy and Breastfeeding

Although the specific mechanism of action of onabotA in migraine is not fully understood, botulinum neurotoxin acts locally at the site of injection with minimal systemic distribution. Mechanistically, it inhibits the intraneuronal target SNAP-25 (synaptosomal-associated protein-25 kDa), one of the SNARE proteins critical for vesicular fusion and then neurotransmitter release [35]. Therefore, onabotA affects mainly the neuronal release of acetylcholine but also other neurotransmitters (including CGRP, glutamate, serotonin, and GABA), considering that SNAREs are involved in vesicular release in general [36]. Specifically, its proposed mechanism of action in migraine involves the modulation of pain pathways through effects on neurotransmitters involved in nociceptive signaling and not the inhibition of muscle contraction [37]. Its large molecular size (~150 kDa for the toxin itself and ~900 kDa the whole molecular complex) justifies the limited systemic absorption after local injection, making onabotA activity predominantly confined to the injection site [38,39]. Furthermore, although at a standard dose for local injections onabotA is not expected to be found in the circulation, even if it passed, it could not cross the placental barrier entering the fetus due to the large molecular size [40].

When onabotA (4, 8 and 16 Units/kg) was administered twice (IM) in pregnant mice and rats during the organogenesis period, reduction in fetal body weight and decreased fetal skeletal ossification were observed in the offspring at the two higher doses. Similarly, other studies that evaluated the administration of onabotA daily (IM) in pregnant rats and rabbits (at different increasing dosages) confirmed the negative fetal outcomes [41].

However, single IM injections (1, 4, or 16 U/kg) at three different periods of development demonstrated no adverse events on fetal development in rats [41]. To note, the adverse effects observed in the above-mentioned preclinical studies showed that they were dose-dependent and primarily observed at dosages able to induce systemic toxicity in the mother. To note, the dosages used are higher that typical human therapeutic doses.

Furthermore, the large molecular size of onabotA and its mechanism of action suggest that it does not transfer into breast milk during breastfeeding or affect the nursing infant; however, clinical studies to confirm this are lacking.

The lack of systemic distribution in standard clinical applications of onabotA may suggest a minimal risk of fetal exposure at therapeutic doses, though human placental transfer data remain limited. Therefore, these findings underline a cautious use of onabotA in pregnancy, avoiding unnecessary exposure, particularly during the first trimester, that is, the organogenesis period.

## 4. Conclusions

Current evidence suggests that onabotA, when used at therapeutic doses for CM prophylaxis, does not appear to significantly increase the risk of major fetal malformations or adverse pregnancy based on a growing body of observational data in humans. Its local mechanism of action and minimal systemic distribution reduce potential risks to both the fetus and the breastfeeding newborn. However, due to the lack of robust data, particularly from controlled clinical trials, its use during pregnancy and breastfeeding should be carefully considered on a case-by-case basis, weighing maternal benefits against potential risks. It is desirable that future studies and numerous reports are conducted to establish definitive safety guidelines regarding the use of onabotA during pregnancy and breastfeeding.

## 5. Materials and Methods

A comprehensive literature search was conducted across multiple electronic databases including PubMed, Scopus, Web of Science, and Cochrane Library. The search was conducted from database inception until first September 2024. Search terms included different combinations of “migraine”, “onabotulinumtoxinA”, “BoNT-A”, “pregnancy”, “breastfeeding”, “lactation”, “chronic migraine”, “teratogenic effects”, and “fetal development”. Additionally, reference lists of relevant articles were manually searched to identify any further studies.

The research question was: “What evidence exists regarding the use of onabotA during pregnancy and breastfeeding for women with chronic migraine?”

The PICO (Population, Intervention, Comparison, Outcome) framework was used for structuring the inclusion criteria for this study:

- Population (P): pregnant or breastfeeding women diagnosed with CM;
- Intervention (I): onabotA injections administered as a preventive migraine therapy (any protocol);
- Comparison (C): standard migraine management approaches (if any) or no treatment;
- Outcomes (O): Maternal and fetal safety, breastfeeding outcomes, and associated risks. Effectiveness in pregnant and breastfeeding women.

RCTs, cohort studies, case series, case reports, and observational studies were included if available. Studies that discussed any phase of pregnancy or breastfeeding (e.g., early or late pregnancy, exclusive or partial breastfeeding) were considered. Inclusion criteria required studies to address onabotA’s effects on migraine management or provide safety data specific to maternal or fetal outcomes. Studies were excluded if they were not available in English, or were reviews, editorials, or opinion papers without primary data.

Data extracted from studies included, if available, study characteristics (author, year, study design, and sample size), patient demographics (age, pregnancy/breastfeeding status, and migraine severity), treatment details, and clinical outcomes (efficacy and adverse events).

Finally, relevant studies including the use of onabotA in pregnancy and breastfeeding in other indications than CM have been briefly reported to provide a complete overview on the topic. Due to the included studies, no formal comparison with other migraine treatments was possible.

## Figures and Tables

**Table 1:** Details of the studies including onabotA for the preventive treatment of chronic migraine.

Diagnosis	Number of Patients	Age or Age Range [years]	Treatment Duration/Occurrence	AEs/SAEs	Pregnancy	Breastfeeding	Reference
CM	1	26	3 months	No significant adverse effects	Healthy babies with normal birth weights and no congenital malformations spanning the 6.5 years of the baby’s life.	N/A	[18]
CM (35.3%), other indications	397 (195 pregnancies, 197 fetuses)	46.5 (10.1)	Approximately 95% of exposure occurred during the first trimester and most (83.5%) were exposed to <200 U.	The prevalence rate of major fetal defects among live births is consistent with the rates reported in the general population.	152 (77.2%) prospective live births and 45 (22.8%) losses: 32 spontaneous and 13 electives. Of the 152 live births, 148 (97.4%) had normal outcomes, while 4 had abnormalities, including 1 major birth defect, 2 minor fetal defects, and 1 birth complication.	N/A	[19]
CM	45 (32 continued treatment)	30.9 (19–42)	Within 3 months prior to conception and during pregnancy. Patients continuing injections followed the original 12 weekly cycles of injections.	No significant adverse effects.	Among the 45 patients, 32 continued treatments during pregnancy, while 13 chose to stop. All but one (miscarriage) of the pregnancies resulted in full-term, healthy babies with normal birth weights and no congenital malformations.	N/A	[20]

## Data Availability

No new data were created or analyzed in this study.
